# Gender Disparities in Hospitalization Outcomes and Healthcare Utilization Among Patients with Systemic Lupus Erythematosus in the United States

**DOI:** 10.7759/cureus.41254

**Published:** 2023-07-01

**Authors:** Fidelis Uwumiro, Victory O Okpujie, Osasumwen Osemwota, Nnenna E Okafor, Michael I Otu, Azabi Borowa, Pascal Ezerioha, Ejiroghene Tejere, Hillary Alemenzohu, Michael M Bojerenu

**Affiliations:** 1 Family Medicine, Our Lady of Apostles Hospital, Akwanga, NGA; 2 Internal Medicine, Central Hospital Benin, Benin City, NGA; 3 Internal Medicine, Department of Health Sciences and Social Work, Western Illinois University, Macomb, USA; 4 Internal Medicine, All Saints University, College of Medicine, Kingstown, VCT; 5 Medicine, University of Calabar, Calabar, NGA; 6 Internal Medicine, College of Medicine, University of Lagos, Lagos, NGA; 7 Internal Medicine, Nnamdi Azikiwe University, Awka, NGA; 8 Internal Medicine, Kharkiv National Medical University, Kharkiv, UKR; 9 Internal Medicine, College of Medicine, University of Ibadan, Ibadan, NGA; 10 Internal Medicine, St. Barnabas Hospital (SBH) Heath System, New York, USA

**Keywords:** unequal access to health care, nationwide inpatient sample (nis), gender disparities, morbidity and mortality, systemic lupus erythematosis

## Abstract

Background

Systemic lupus erythematosus (SLE) is a multisystem autoimmune disease characterized by various clinical manifestations. Despite efforts to improve outcomes, mortality rates remain high, and certain disparities, including gender, may influence prognosis and mortality rates in SLE. This study aims to examine the gender disparities in outcomes of SLE hospitalizations in the US.

Methods

We conducted a retrospective analysis of the Nationwide Inpatient Sample (NIS) database between 2016 and 2020. The NIS database is the largest publicly available all-payer database for inpatient care in the United States, representing approximately 20% of all hospitalizations nationwide. We selected every other year during the study period and included hospitalizations of adult patients (≥18 years old) with a primary or secondary diagnosis of SLE using International Classification of Diseases, Tenth Revision (ICD-10) codes. The control population consisted of all adult hospitalizations. Multivariate logistic regression was used to estimate the strength of the association between gender and primary and secondary outcomes. The regression models were adjusted for various factors, including age, race, median household income based on patients' zip codes, Charlson comorbidity index score, insurance status, hospital location, region, bed size, and teaching status. To ensure comparability across the years, revised trend weights were applied as the healthcare cost and use project website recommends. Stata version 17 (StataCorp LLC, TX, USA) was used for the statistical analyses, and a two-sided P-value of less than 0.05 was considered statistically significant.

Results

Among the 42,875 SLE hospitalizations analyzed, women accounted for a significantly higher proportion (86.4%) compared to men (13.6%). The age distribution varied, with the majority of female admissions falling within the 30- to 60-year age range, while most male admissions fell within the 15- to 30-year age category. Racial composition showed a slightly higher percentage of White Americans in the male cohort compared to the female cohort. Notably, more Black females were admitted for SLE compared to Black males. Male SLE patients had a higher burden of comorbidities and were more likely to have Medicare and private insurance, while a higher percentage of women were uninsured. The mortality rate during the index hospitalization was slightly higher for men (1.3%) compared to women (1.1%), but after adjusting for various factors, there was no statistically significant gender disparity in the likelihood of mortality (adjusted odds ratio (aOR): 1.027; 95% confidence interval (CI): 0.570-1.852; P=0.929). Men had longer hospital stays and incurred higher average hospital costs compared to women (mean length of stay (LOS): seven days vs. six days; $79,751 ± $5,954 vs. $70,405 ± $1,618 respectively). Female SLE hospitalizations were associated with a higher likelihood of delirium, psychosis, and seizures while showing lower odds of hematological and renal diseases compared to men.

Conclusion

While women constitute the majority of SLE hospitalizations, men with SLE tend to have a higher burden of comorbidities and are more likely to have Medicare and private insurance. Additionally, men had longer hospital stays and incurred higher average hospital costs. However, there was no significant gender disparity in the likelihood of mortality after accounting for various factors.

## Introduction

Systemic lupus erythematosus (SLE) is a chronic autoimmune disease characterized by widespread inflammation that can affect multiple organ systems [1,2]. This complex condition is reported to predominantly affect women, with a significantly higher prevalence compared to men [3,4]. While the gender bias in SLE is well-established, the extent to which gender influences hospitalizations and inpatient outcomes, particularly mortality, among patients with SLE remains an area of vital research and inquiry. Understanding these disparities is important for ensuring equitable healthcare provision and improving outcomes within this patient population.

The index study presents the findings of an extensive nationwide population-based study aimed at examining the profound impact of gender disparities on hospitalizations and inpatient outcomes for patients with SLE in the United States, with a particular focus on mortality and other critical outcome measures. By harnessing a vast and diverse dataset, the study aims to assess the contribution of gender to differential outcomes within the context of SLE.

Beyond well-documented epidemiological differences, including disease prevalence and age of onset, gender is believed to exert a significant influence on disease management, treatment response, and long-term prognosis in SLE [5]. Existing research has hinted at the likelihood of more severe disease manifestations, higher disease activity, and increased disease-related damage among females with SLE compared to males [6-8]. However, limited attention has been given to the role of gender disparities in hospitalization rates and subsequent outcomes within the SLE population, particularly concerning mortality and other crucial measures.

In this study, we examined a large, representative cohort of patients diagnosed with SLE, spanning diverse geographic regions. We aim to unravel the specific dimensions of gender disparities in hospitalizations and inpatient outcomes within this population, with a particular emphasis on mortality as a critical outcome measure. Our investigation encompasses an assessment of outcomes such as hospitalization rates, length of stay, complications, and mortality, all stratified by gender.

The findings from this study have potential implications for clinical practice, healthcare policies, and public health interventions. By identifying and quantifying the impact of gender on hospitalizations and inpatient outcomes, particularly mortality, we can devise targeted strategies to optimize care delivery, allocate resources effectively, and enhance patient outcomes for both male and female individuals living with SLE.

## Materials and methods

The Nationwide Inpatient Sample Database (NIS)

We conducted a retrospective cohort study utilizing data from the Nationwide Inpatient Sample (NIS) database spanning the years 2016 to 2020. The NIS database stands as the largest all-payer inpatient dataset, providing discharge data that approximate national figures in the United States [9,10]. The NIS database is a robust and widely utilized resource in healthcare research, providing valuable insights into inpatient hospitalizations across the United States. It encompasses a representative sample of discharges from community hospitals, including academic medical centers, teaching hospitals, and non-teaching facilities. With its extensive coverage spanning diverse geographic regions, the NIS offers a comprehensive snapshot of the nation's inpatient healthcare landscape. Its wealth of data encompasses a wide range of clinical, demographic, and utilization variables, allowing researchers to explore various aspects of healthcare delivery, disease patterns, and outcomes. The meticulous design employed in constructing the NIS ensures data accuracy, reliability, and generalizability, empowering researchers to derive meaningful and evidence-based conclusions to inform healthcare policies, improve patient care, and advance medical knowledge.

Inclusion criteria and study variables

This study included all hospital admissions of individuals aged 15 years and older (reproductive age group) whose primary discharge diagnosis was SLE. The primary diagnosis, all confounders, and complications of SLE were defined using the International Classification of Diseases Tenth Revision Clinical Modification and Procedure Coding System (ICD-10-CM/PCS). The total cohort was dichotomized based on gender into male and female categories, while age categories were defined as 15-30, 31-60, and >60 years. Hospitalizations with missing data or incomplete records were excluded from the study.

Ethical considerations

Institutional Review Board approval was not required because the database used for this study is de-identified, publicly available, and does not include protected healthcare information.

Outcome measures

The primary outcome of interest was inpatient mortality, while secondary outcomes included the mean length of hospitalization, the mean total hospital charges, and the likelihood of experiencing SLE complications between males and females. Mortality is recorded within a dichotomous variable (DIED) in the NIS. The mortality variable is recorded as one (died during the index admission) or 0 (did not die during the index admission). The length of stay and hospital charges variables are recorded as numerical variables, allowing for quantitative analysis such as calculating averages, performing statistical tests, and examining trends or patterns in hospital charges.

Statistical analysis

Data analysis was performed using Stata version 17 software (StataCorp LLC, Texas, USA). Sample weighting was applied during analyses to ensure compliance with the Healthcare Cost and Utilization Project regulations concerning the use of the NIS database for generating national estimates. Baseline patient and hospital-level characteristics and comorbidities between the male and female groups were compared using chi-square tests. An initial univariate screen including all variables listed in Table 1 was performed to identify factors related to the outcomes of interest.

**Table 1:** Baseline sociodemographic composition and resource utilization of SLE hospitalizations ^a^Significant at values <0.05 SNF: skilled nursing facility; ICF: intermediate care facility; HMO: health maintenance organization; USD: United States dollar

Variable	Male, (%)	Female, (%)	P-value^a^
Total admissions (42,875)	13.6	86.4	
Mean age, in years	37 ± 0.5	36 ± 0.2	0.697
Age groups			<0.001
15 – 30	44.6	41.4	
31 – 60	41.7	49.9	
>60	13.7	8.7	
Mean hospital charge, USD	79,751 ± 5,954	70,405 ± 1,618	0.003
Aggregate hospital charge, USD	459 million	2.58 billion	<0.001
Elective admission, %	6.7	6.6	0.87
Emergency admission, %	93.3	93.4	0.87
Mean length of hospital stay, days	7 ± 0.3	6 ± 0.1	<0.001
Discharge disposition, %			<0.001
Died in the hospital	1.3	1.1	
Routine home discharge	79.4	79.4	
Transfer to a short-term hospital	2.5	2.8	
Transfer to other skilled care (Including SNF, home health care & ICF)	14.3	14.7	
Against medical advice	2.8	2.3	
Discharge quarter, %			0.101
First quarter	22.9	24.1	
Second quarter	23.22	25.1	
Third quarter	26.31	26.4	
Fourth quarter	27.6	24.4	
Race, %			<0.001
White American	27	22.4	
Black	36.4	46.9	
Hispanic	25.8	21.2	
Asian or Pacific Islander	5.8	4.9	
Native American	0.6	0.6	
Others	4.4	4.1	
Weekend admission, %	20.7	21.7	
Combined Charlson comorbidity index, %			<0.001
0	0.9	1	
1	40	44.8	
2	13.8	17.9	
≥3	45.2	36.3	
Median annual income quartiles, %			0.67
1–43,999	37.6	38.7	
44,000–55,999	24.5	24.7	
56,000–73,999	21.3	21.5	
≥ 74,000	16.6	15.2	
Insurance status, %			0.005
Medicare	22.6	24.4	
Medicaid	35.2	36.6	
Private, including HMO	33.3	32.9	
Self-pay	9	6.2	
Hospital characteristics, %			0.471
Rural	2.2	2.9	
Urban	12.3	14.2	
Teaching hospital	85.5	82.9	
Northeast	20.4	20.3	
Midwest	18.8	16.5	
South	39.5	42.8	
West	21.3	20.3	
Small	13.3	14.8	
Medium	25.4	24.8	
Large	61.3	60.4	

The association between outcomes and gender was subsequently assessed using multivariate regression analyses, including relevant variables with a p-value less than 0.1 on the univariate screen. The likelihood of mortality was calculated as an adjusted odds ratio, while the secondary outcomes were calculated as an adjusted mean difference. The threshold for statistical significance was set at a p-value of less than 0.05 for all analyses.

## Results

Sociodemographic characteristics

The analysis utilized the NIS combined database spanning from 2016 to 2022, consisting of over 139 million weighted hospital discharges. From this vast dataset, a total of 42,875 adult hospitalizations with systemic lupus erythematosus (SLE) as the primary diagnosis were selected for the study. Among these cases, 5,831 admissions (13.6%) were attributed to men, while 37,044 (86.4%) were attributed to women.

A comprehensive overview of the sociodemographic characteristics and resource utilization of the study population is presented in Table 1. The mean age of both male and female patients in the study was comparable (37 years for men and 36 years for women). However, the two cohorts had notable differences in the age distribution. The majority of female admissions fell within the age range of 30 to 60 years (49.9%), whereas the majority of male admissions fell within the 15 to 30 age category (44.6%). Additionally, a noteworthy proportion (13%) of SLE admissions among males were for individuals aged over 60, compared to 8.7% in the female cohort (P<0.001).

Regarding racial composition, White Americans accounted for 27% of the male SLE population, slightly higher than the 22.4% observed in the female cohort. Conversely, more Black females were admitted for SLE than Black males (46.9% vs. 36.4%; P<0.001). The male population exhibited a higher burden of comorbidities, with approximately 45.2% of males having a Charlson comorbidity index score of 3 or greater, compared to 36.3% in the female cohort. In terms of insurance coverage, a greater proportion of men had Medicare and private insurance, while a higher percentage of women were uninsured (9% vs. 6.2%). These findings are summarized in Table 1.

Primary outcome: mortality

During the index hospitalization, the mortality rate among men admitted for SLE was 1.3%, slightly higher than the rate of 1.1% observed in the female population. However, after accounting for various patient and hospital-level factors, a comprehensive multivariate analysis revealed no statistically significant gender disparity in the likelihood of mortality (adjusted odds ratio (aOR): 1.027; 95% confidence interval (CI): 0.570-1.852; P=0.929) (Table 2).

**Table 2:** Adjusted odds ratio of outcomes and complications for female vs. male SLE hospitalizations on multivariate regression analysis **adjusted mean difference USD: United States dollar; aOR: adjusted odds ratio; CI: confidence interval; SLE: systemic lupus erythematosus #Statistically significant

Outcome	aOR (95% CI)	P-value
Primary outcome		
In-hospital mortality	1.027 (0.570-1.852)	0.929
Secondary outcomes		
Mean length of hospital stay	-0.41^**^ (-0.979 to 0.159)	0.158
Mean total hospital charges, USD	-5316^**^ (-18182 to 7,549)	0.418
Complications of SLE		
Hematological disorders	0.819 (0.689-0.975)	0.024^#^
Immunological disorders	1.741 (0.522-5.812)	0.367
Oral ulcers	3.966 (0.532-29.585)	0.179
Renal disease	0.701 (0.559-0.879)	0.002^#^
Seizures	1.839 (1.306-2.591)	<0.001^#^
Alopecia	0.712 (0.202-2.508)	0.597
Serositis	0.855 (0.387-1.893)	0.700
Delirium/psychosis	1.564 (1.184-2.067)	0.002^#^
Discoid rash	0.787 (0.179-3.466)	0.751
Acute cutaneous lupus	0.869 (0.668-1.130)	0.294
Chronic cutaneous lupus	1.871 (0.561-6.242)	0.308
Pulmonary embolism	0.599 (0.344-1.043)	0.070
Acute coronary syndromes	0.833 (0.418-1.661)	0.604
Pulmonary hypertension	1.048 (0.708-1.551)	0.816
Peptic ulceration	1.801 (0.408-7.944)	0.437
Interstitial lung disease	0.971 (0.673-1.399)	0.873
Endocarditis	1.654 (0.479-5.709)	0.426
Pancreatitis	1.054 (0.609-1.822)	0.852

Secondary outcomes

Men were admitted for longer periods compared to females (mean length of stay (LOS): seven days vs. six days for female admissions) and paid more in average hospital costs compared to females ($79,751 ± $5,954 vs. $70,405 ± $1,618 respectively). However, there was no significant difference in the mean length of hospital stay or mean hospital charges between them (Table 2).

SLE hospitalizations for female patients were found to be associated with an increased likelihood of delirium, psychosis (aOR: 1.564; 95% CI: 1.184-2.067; P=0.002), and seizures (aOR: 1.839; 95% CI: 1.306-2.591; P< 0.001) and lower odds of hematological (aOR: 0.819; 95% CI: 0.689-0.975; P=0.024) and renal diseases (aOR: 0.701; 95% CI: 0.559-0.879; P=0.002) (Table 2).

## Discussion

The findings of the index study reveal distinct sociodemographic characteristics between male and female SLE patients. While the mean age of both groups was comparable, there were notable differences in the age distribution. Females comprised the majority of SLE admissions within the age range of 30 to 60 years, whereas males had a higher proportion of admissions in the 15 to 30 age category. Moreover, a significant percentage of male SLE admissions were among individuals aged over 60, compared to the female cohort. These age-related differences have important clinical implications. Younger male SLE patients may require increased attention and targeted interventions to address disease manifestations and complications specific to this age group [11,12]. Additionally, the higher proportion of older males with SLE highlights the need for comprehensive management strategies that consider age-related comorbidities and treatment interactions [13].

Racial disparities were also observed, with White Americans accounting for a slightly higher proportion of male SLE admissions compared to females. Conversely, a greater number of Black females were admitted for SLE compared to Black males. These racial and gender disparities in SLE hospitalizations warrant further investigation into the underlying factors contributing to these disparities. Tailored approaches that consider the unique needs and challenges faced by different racial and gender groups can help improve SLE outcomes and reduce disparities [14,15]. The burden of comorbidities and insurance coverage showed some gender differences, with males carrying a higher burden of comorbidities and a greater percentage having Medicare and private insurance. Females, on the other hand, had a higher percentage of uninsured individuals. These findings emphasize the importance of comprehensive care coordination and access to healthcare services for all SLE patients, regardless of gender [16]. Addressing the disparities in comorbidities and insurance coverage can enhance overall disease management and improve outcomes [17,18].

The primary outcome of interest in this study was inpatient mortality among SLE admissions. The analysis revealed a slightly higher mortality rate among males compared to females during the index hospitalization. However, after adjusting for various patient and hospital-level factors, no statistically significant gender disparity in the likelihood of mortality was observed. These findings suggest that while there may be initial differences in mortality rates between male and female SLE patients, other factors, such as disease severity, comorbidities, and access to care, may contribute more significantly to mortality risks [19-21]. This underscores the need for comprehensive assessment and management of these factors to improve survival outcomes for all SLE patients.

The secondary outcomes of this study focused on the length of hospital stay, hospital charges, and the likelihood of SLE-related complications. Male SLE patients had longer hospital stays compared to females, accompanied by higher average hospital costs. Although the differences were not statistically significant, these findings suggest the need for closer monitoring and management of male SLE patients during hospitalization to ensure timely and efficient care. Additionally, healthcare providers should be mindful of potential financial burdens on patients and explore strategies to mitigate costs without compromising the quality of care.

In terms of complications related to SLE, females exhibited a higher probability of experiencing delirium, psychosis, and seizures compared to males. On the other hand, females had lower chances of developing hematological and renal diseases. These findings contrast with previous studies that have documented higher seizure rates in men [22]. The observed gender differences in complications suggest a potential shift in the prevailing trends and underscore the significance of tailored approaches to managing and monitoring these specific complications. Healthcare providers should remain vigilant in detecting and addressing these complications, particularly in female SLE patients, to enhance outcomes and improve their overall quality of life.

The findings of this study have several clinical implications. Firstly, understanding the sociodemographic characteristics of SLE patients, such as gender, age, and racial disparities, can inform healthcare providers in tailoring their approaches to address the specific needs and challenges faced by different patient populations. Comprehensive care plans that consider age-related comorbidities, disease manifestations, and treatment interactions can improve patient outcomes and quality of life. Secondly, the absence of a significant gender disparity in mortality rates after adjusting for confounding factors suggests that other factors play a more substantial role in determining mortality risks in SLE. Identifying and addressing these factors, including disease severity, comorbidities, and access to care, is crucial for reducing mortality rates in both male and female SLE patients. Lastly, the gender differences in SLE-related complications highlight the importance of tailored management approaches. Healthcare providers should be vigilant in recognizing and managing complications such as delirium, psychosis, and seizures, as well as hematological and renal diseases, especially among female SLE patients. Early detection and targeted interventions can help minimize the impact of these complications and improve overall patient outcomes.

The study has some limitations related to the use of administrative databases. These limitations include non-randomization and inadequate records of disease severity, which could potentially impact the assessment of mortality. Additionally, owing to the nature of the NIS, the identification of comorbidities in this study was conducted without utilizing admission indicators to differentiate between pre-existing comorbid conditions and complications that arise during hospitalization, potentially resulting in a significant overlap between comorbidity and complications.

## Conclusions

In conclusion, this study demonstrates that gender disparities exist in sociodemographic characteristics, insurance status, inpatient complications, and resource utilization among patients admitted with SLE. The findings highlight the need for tailored approaches that consider the sociodemographic characteristics, burden of comorbidities, and insurance coverage of SLE patients. Addressing these disparities and implementing targeted interventions can lead to improved outcomes, reduced complications, and enhanced quality of care for all SLE patients, irrespective of their gender. Further research is needed to explore the underlying mechanisms contributing to gender disparities in SLE outcomes and to develop evidence-based strategies for mitigating these disparities and optimizing patient care.
